# HDAC11-Mediated Deacetylation of Triosephosphate Isomerase 1 Promotes Idiopathic Pulmonary Fibrosis

**DOI:** 10.34133/research.0953

**Published:** 2025-10-16

**Authors:** Yu Li, Xiangguang Shi, Feiyang Zhang, Xiumin Zhou, Xinyu Zhu, Jiawei Chen, Kai Fu, Jun Chen, Jian Yang, Zhike Chen, Xin Tong, Jun Zhao, Chang Li

**Affiliations:** ^1^ Department of Thoracic Surgery, The First Affiliated Hospital of Soochow University, Suzhou, China.; ^2^ Institute of Thoracic Surgery, The First Affiliated Hospital of Soochow University, Suzhou, China.; ^3^ Institute of Minimally Invasive Thoracic Cancer Therapy and Translational Research, Suzhou Medical College of Soochow University, Suzhou 215000, Jiangsu, China.; ^4^ Department of Dermatology, Huashan Hospital, Fudan University, Shanghai, China.; ^5^ Suzhou Medical College of Soochow University, Suzhou, China.; ^6^ Department of Oncology, The First Affiliated Hospital of Soochow University, Suzhou, China.; ^7^ Department of Thoracic Surgery, Changshu Hospital Affiliated to Nanjing University of Chinese Medicine, Changshu, China.

## Abstract

Idiopathic pulmonary fibrosis (IPF) is a type of chronic progressive fibrotic interstitial pneumonia and has a poor prognosis due to the lack of effective treatments. Despite extensive investigations into its molecular and cellular mechanisms, the regulatory mechanism involved remains incompletely understood. Triosephosphate isomerase 1 (TPI1), an enzyme in the glycolytic pathway, has emerged as a key research focus in oncogenesis due to its multifaceted roles in malignant progression. However, its role in IPF has not yet been reported. Here, we report that TPI1 expression was elevated in IPF tissues and in mice with bleomycin-induced pulmonary fibrosis. TPI1 knockdown attenuated IPF progression in vitro and in vivo. Mechanistically, we found that histone deacetylase 11 (HDAC11)-mediated deacetylation of TPI1 K69 was enhanced by transforming growth factor-beta1. Deacetylation of TPI1 K69 enhanced its protein stability by attenuating K48-linked polyubiquitination, which enhanced fibroblast-to-myofibroblast differentiation, cell proliferation, and migration. Notably, we designed and tested the activity of a novel cell-penetrating peptide that increased the acetylation of TPI1 and markedly promoted TPI1 degradation, thereby effectively reducing fibrosis. Together, our findings revealed that targeting TPI1 acetylation is an effective strategy for IPF therapy, and the specific cell-penetrating peptide could prevent IPF by promoting the acetylation of TPI1.

## Introduction

Idiopathic pulmonary fibrosis (IPF) is a progressive interstitial lung disease marked by irreversible structural remodeling of the lung. Abnormal alterations in signaling pathways, including the transforming growth factor-beta (TGF-β) signaling pathways, lead to the deposition of collagen in the lung interstitial and alveolar structures, causing damage to the normal lung architecture [1–3]. The incidence of IPF ranges from approximately 0.09 to 4.51 cases per 10,000 persons [4–6]. The median survival time for patients diagnosed with IPF is only 2 to 4 years [7,8]. Few treatment options exist for IPF, and only pirfenidone and nintedanib have been approved for the treatment of IPF [9]. Therefore, understanding the molecular pathogenic mechanisms of IPF and developing novel therapeutic strategies are crucial for prolonging the survival of IPF patients.

Metabolic disorders are implicated in the pathogenesis of many diseases, such as diabetes [10], inflammasome disorders [11], and cancers [12]. Recent studies have highlighted triosephosphate isomerase 1 (TPI1), a key glycolytic enzyme catalyzing the interconversion of dihydroxyacetone phosphate and glyceraldehyde 3-phosphate, as a potential regulator of disease progression. TPI1 dysfunction has been associated with metabolic reprogramming or abnormal activation of signaling pathways in cancer metastasis, but its role in fibrotic diseases remains unexplored. However, there is little information on the role of cellular metabolism in fibrosis. The uptake of glucose is increased in fibroblastic foci [13,14], transforming growth factor-beta1 (TGF-β1) induces the enhancement of glycolysis, and blocking glycolytic flux suppresses TGF-β1-induced fibrosis [15], suggesting that normalizing glycolysis regulation may represent a novel strategy for antifibrotic therapy. Notably, as a central enzyme maintaining glycolytic flux, TPI1 may serve as a critical node connecting metabolic alterations with fibrotic remodeling. Recent studies have highlighted the importance of posttranslational modification [16–19], such as acetylation, in modulating cellular metabolism [20,21] and signaling pathways [22,23]. Acetylation, a key posttranslational modification, serves as a critical coordinator of metabolic enzyme function, including those involved in glycolysis [24]. Acetylation of glycolytic enzymes can alter their catalytic activity, subcellular localization, and protein stability, thereby impacting glycolytic flux [25]. Dysregulated acetylation may influence glycolysis and contribute to fibrotic progression by modulating the metabolic reprogramming of myofibroblasts. This regulatory mechanism may be particularly relevant for TPI1, given its pivotal position in the glycolytic pathway.

In this study, we identified TPI1 as a key factor regulating IPF progression. TPI1 K69 is deacetylated by histone deacetylase 11 (HDAC11), which decreases TPI1 K48-linked polyubiquitination and results in TPI1 accumulation. Cell-penetrating peptides (CPPs) enable efficient cellular entry by interacting with the cell membrane through electrostatic or hydrophobic forces. They often utilize endocytosis or direct translocation to deliver cargo, such as peptides or nucleic acids, into cells. Here, we targeted the K69 site of TPI1 with a CPP, which enhanced TPI1 acetylation, leading to increased TPI1 degradation and inhibition of pulmonary fibrosis. These findings not only establish TPI1 as a novel regulator of fibrotic progression but also reveal an acetylation-dependent mechanism controlling its stability and pro-fibrotic activity.

## Results

### TPI1 is upregulated in IPF

**Fig. 1.**
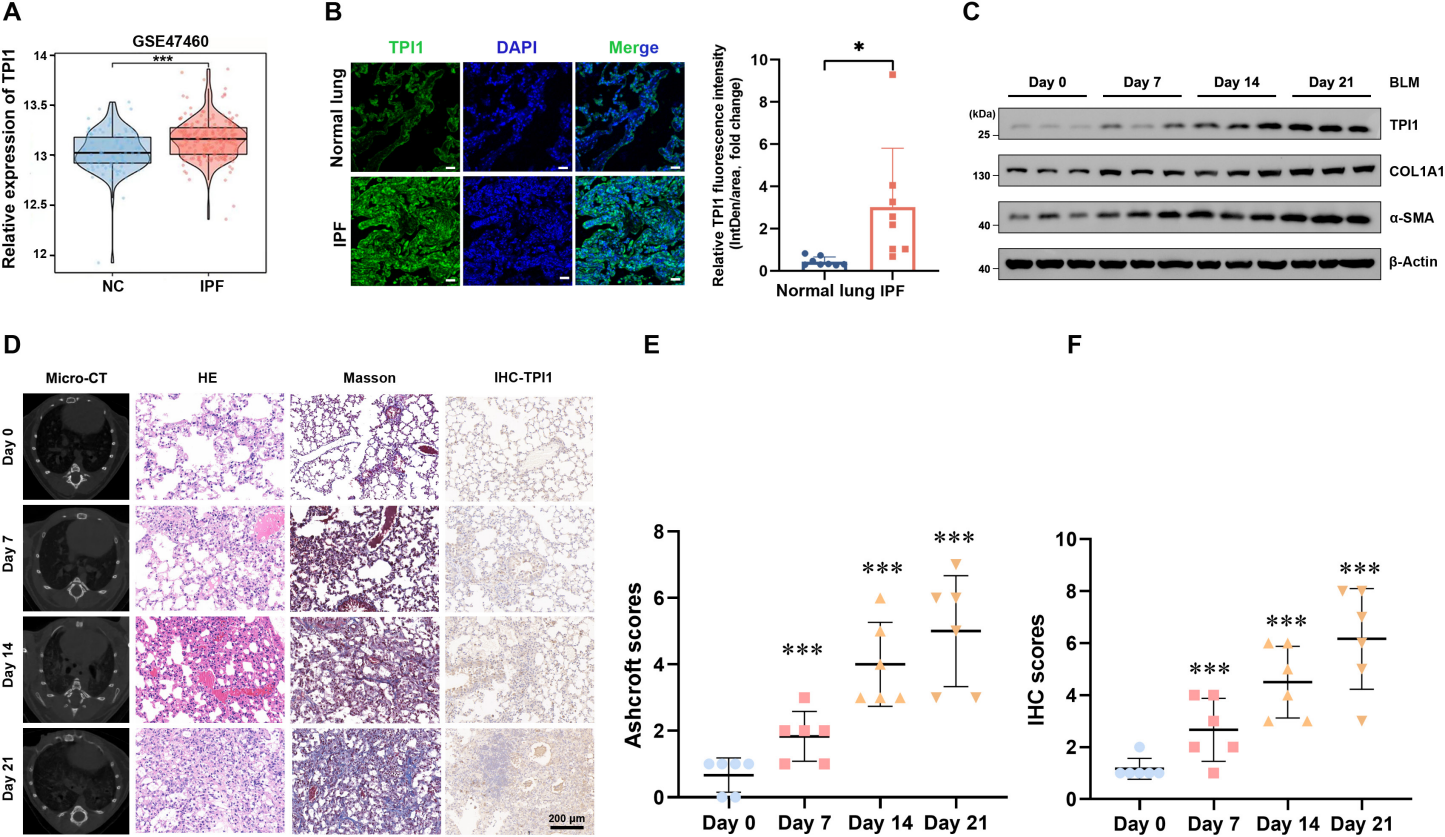
Triosephosphate isomerase 1 (TPI1) is upregulated in idiopathic pulmonary fibrosis (IPF). (A) The GSE47460 dataset from the Gene Expression Omnibus (GEO) database was used to analyze the differences in the messenger RNA (mRNA) levels of TPI1 between the normal control group and the IPF group. ***P < 0.001. (B) Left panel: slides from normal lung and IPF tissues were subjected to multiplex immunofluorescence (multi-IF) staining. TPI1 fluorescence is shown in green, and nuclear fluorescence is shown in blue. Right panel: relative quantitative analysis of the fluorescence intensity of TPI1. The scale bar represents 200 μm; *n* = 3 biological replicates for each analysis. *P < 0.05. (C) Western blot analysis of TPI1, alpha-smooth muscle actin (α-SMA), and collagen type I alpha 1 (COL1A1) expression in the lung tissue of mice; β-actin served as an internal control. (D to F) Left panel: representative images for micro-computed tomography (micro-CT), hematoxylin and eosin (HE) staining, Masson staining, and immunohistochemical (IHC) staining in mice (D). The mice were divided into groups as indicated; the minimal number of mice was used (*n* = 6 per group). The scale bar represents 200 μm. Right panel: analysis of the Ashcroft scores of collagen expression (E) and IHC scores of TPI1 expression (F) in mouse lungs. ***P < 0.001. NC, normal control; IntDen, integrated density; DAPI, 4′,6-diamidino-2-phenylindole; BLM, bleomycin.

As demonstrated in recent studies, it can be concluded that glycolysis has a pivotal function in both the initiation and progression of pulmonary fibrosis [15]. TPI1 primarily catalyzes the isomerization of glyceraldehyde 3-phosphate and dihydroxyacetone phosphate [26], thereby regulating the glycolytic process in cells [27]. However, the role of TPI1-mediated glycolytic dysfunction in the progression of IPF remains to be clarified. The present study aims to investigate the hypothesis that TPI1 is involved in the development of IPF. We firstly analyzed the IPF patient dataset GSE47460 from the Gene Expression Omnibus database. Our analysis revealed that TPI1 was upregulated in many IPF tissues (Fig. 1A). Next, we collected lung tissues from IPF patients and performed a multiplex immunofluorescence analysis to examine the expression of TPI1 in IPF and healthy lung tissue. We found that there was a significant increase in TPI1 expression in IPF lung tissues compared to that in normal lung tissues (Fig. 1B). To further validate these findings, we examined TPI1 expression in a bleomycin (BLM)-induced mouse model of IPF. Consistent with the patient data, we observed a time-dependent upregulation of TPI1 in the fibrotic lung tissues of BLM-treated mice, with expression levels progressively increasing at 7, 14, and 21 d post-induction. Notably, the expressions of fibrosis markers collagen type I alpha 1 (COL1A1) and alpha-smooth muscle actin (α-SMA) exhibited a parallel gradient elevation over the same time course, closely correlating with TPI1 dynamics (Fig. 1C). Micro-computed tomography (micro-CT) and hematoxylin and eosin (HE) staining revealed that lung injury and fibrosis increased over time in mice. Meanwhile, Masson staining indicated increased collagen deposition (Fig. 1D to F). These results indicated that TPI1 may play a pivotal regulatory role in driving the pathogenesis of IPF.

### TPI1 modulates IPF in vitro and in vivo

**Fig. 2.**
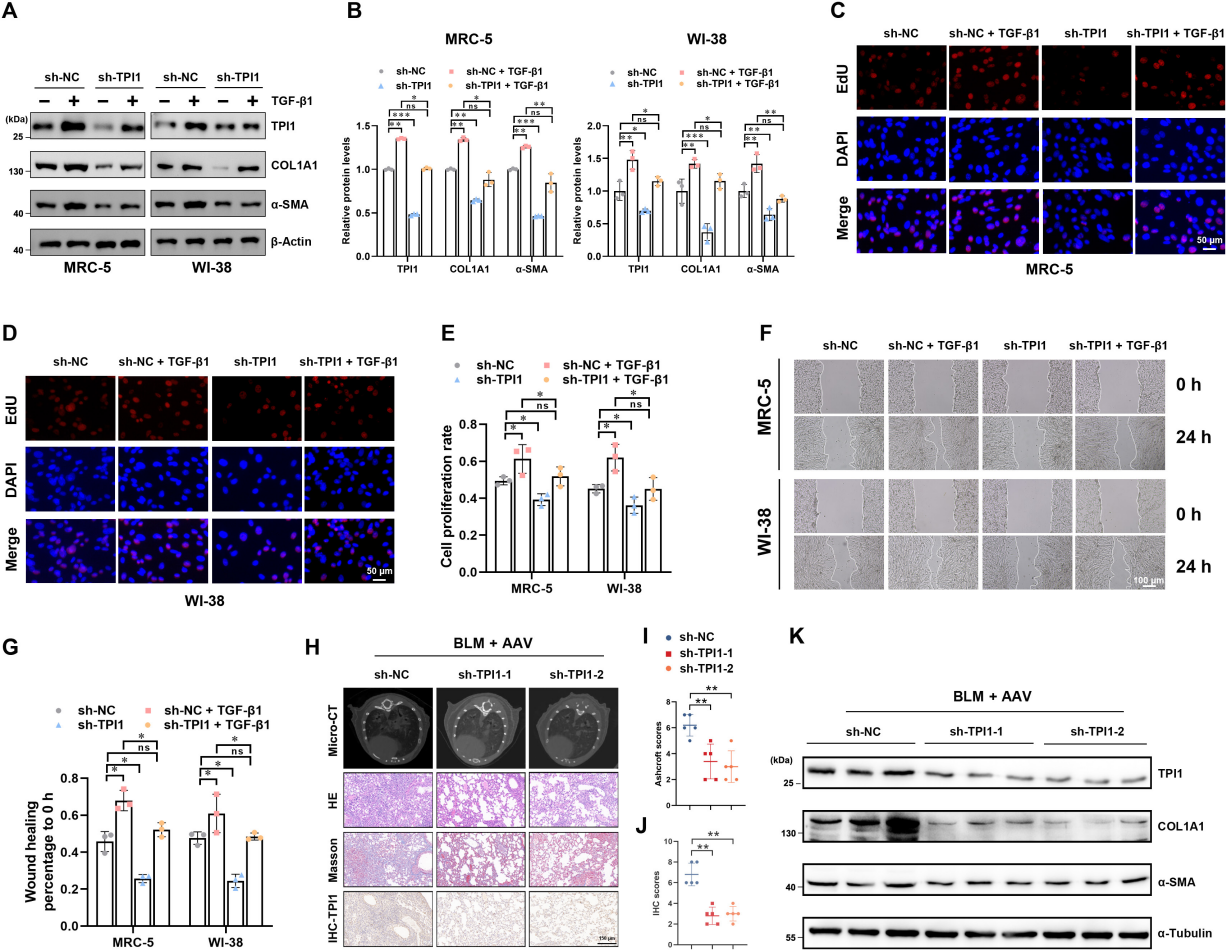
TPI1 modulates IPF in vitro and in vivo. (A and B) TPI1 knockdown in MRC-5 and WI-38 cells pretreated with 5 ng/ml transforming growth factor-beta1 (TGF-β1) for 24 h. Western blot analysis of TPI1, α-SMA, and COL1A1 expression in the indicated cells; β-actin served as an internal control. *P < 0.05; **P < 0.01; ***P < 0.001. (C to E) A 5-ethynyl-2′-deoxyuridine (EdU) assay was performed to evaluate the proliferation of TPI1-knockdown cells pretreated with 5 ng/ml TGF-β1 for 24 h. The percentage of EdU-positive cells was calculated by using ImageJ. The scale bar represents 50 μm; *n* = 3 biological replicates for each analysis. *P < 0.05; ns, no significance. (F and G) A wound healing assay was performed to evaluate cell migration. The extent of wound healing was recorded and quantified by using ImageJ. The scale bar represents 100 μm; *n* = 3 biological replicates for each analysis. *P < 0.05; ns, no significance. (H to J) Left panel: representative images for micro-CT, HE staining, Masson staining, and IHC staining in mice. The mice were divided into groups as indicated; the minimal number of mice was used (*n* = 6 per group). The scale bar represents 150 μm. Right panel: analysis of the IHC scores of TPI1 expression and Ashcroft scores of collagen expression in mouse lungs. **P < 0.01. (K) Western blot analysis of TPI1, α-SMA, and COL1A1 expression in the lung tissue of mice; α-tubulin served as an internal control. AAV, adeno-associated virus.

To explore whether TPI1 is involved in the progression of pulmonary fibrosis, we used TGF-β1 to induce the transformation of fibroblasts into myofibroblasts and then measured fibrosis markers in MRC-5 and WI-38 cells with stable TPI1 silencing. Our results revealed that TPI1 knockdown significantly decreased COL1A1 and α-SMA protein in cell lines that had been pretreated with TGF-β1 (Fig. 2A and B), indicating that TPI1 suppression inhibits the transformation of fibroblasts into myofibroblasts. In addition, 5-ethynyl-2′-deoxyuridine (EdU) staining and wound healing assays demonstrated that silencing TPI1 reduced the proliferation (Fig. 2C to E) and migration (Fig. 2F and G) of MRC-5 and WI-38 cells. In contrast, TPI1 overexpression significantly enhanced the transformation of fibroblasts into myofibroblasts (Fig. S1A and B) and the proliferation (Fig. S1C to E) and migration (Fig. S1F and G) of MRC-5 and WI-38 cells.

To further assess the role of TPI1 in vivo, we generated an adeno-associated virus 6 (AAV-6) virus targeting knockdown TPI1 expression and administered it to mice before inducing fibrosis via BLM treatment. Micro-CT and HE staining showed that lung injury and fibrosis were alleviated in mice with inhibited TPI1 expression. Masson staining indicated reduced collagen deposition (Fig. 2H to J). The protein levels of fibrosis markers COL1A1 and α-SMA were decreased when TPI1 was knocked down (Fig. 2K).

### Acetylation of TPI1 at the K69 residue

**Fig. 3.**
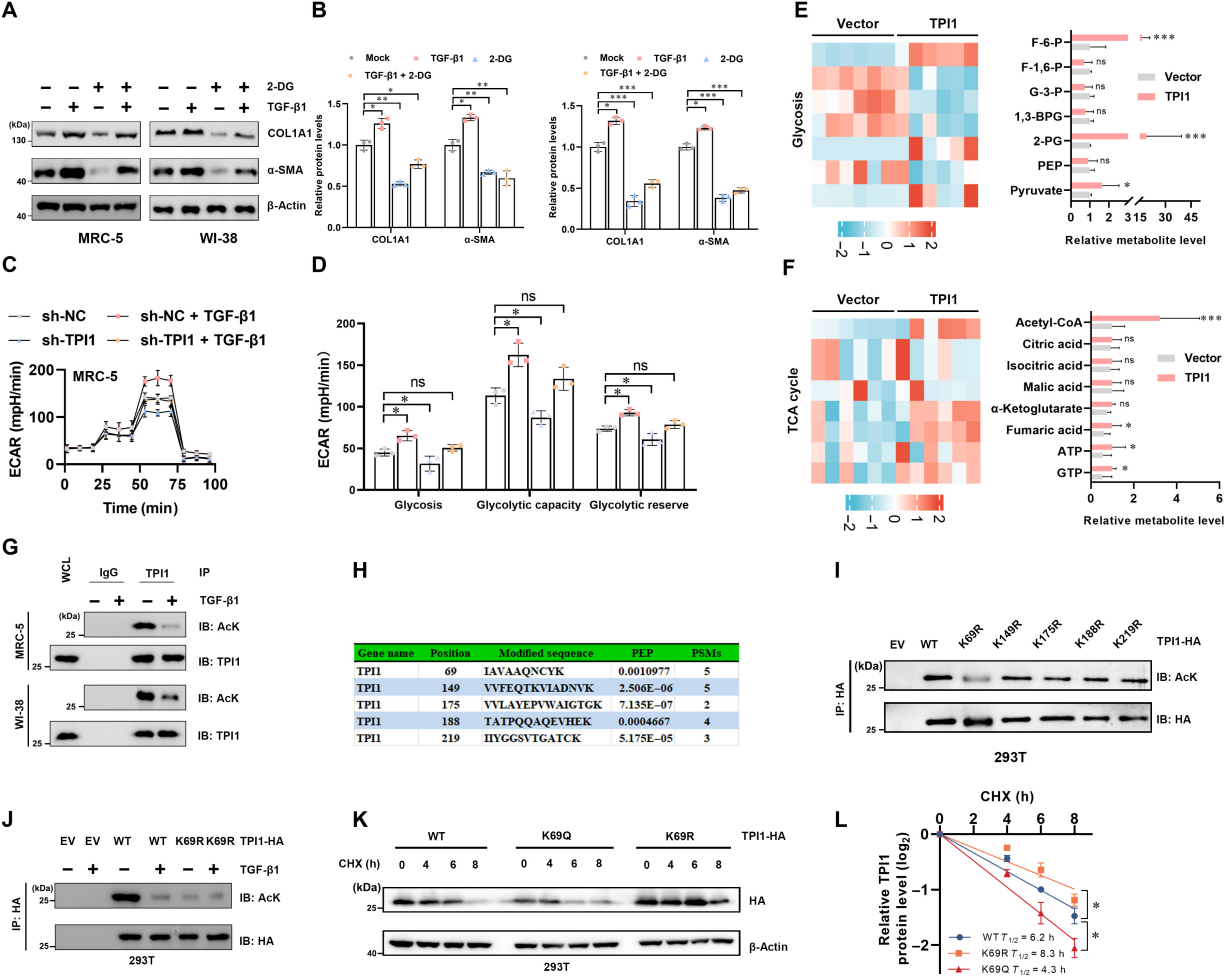
Acetylation of TPI1 at the K69 residue. (A and B) MRC-5 and WI-38 cells were treated with 5 ng/ml TGF-β1 or 10 mM 2-deoxyglucose (2-DG) for 24 h. Western blot analysis of TPI1, α-SMA, and COL1A1 expression in the indicated cells; β-actin served as an internal control. *P < 0.05; **P < 0.01; ***P < 0.001. (C and D) Real-time extracellular acidification rates (ECARs) of MRC-5 cells silencing TPI1 according to a glycolysis stress test and semiquantitative analysis of glycolysis, glycolytic capacity, and the glycolytic reserve. *n* = 3; error bars, means ± SD; all analyses were performed using one-way analysis of variance with Tukey’s post hoc test. *P < 0.05; ns, no significance. (E and F) Heatmaps and quantitative analysis of glycolysis (E) and tricarboxylic acid (TCA) cycle (F) related metabolites (*n* = 6). *P < 0.05; ***P < 0.001. (G) Western blot analysis of whole-cell lysates (WCLs) and anti-TPI1 immunoprecipitates derived from MRC-5 cells and WI-38 cells; 5 ng/ml TGF-β1 as indicated for 4 h before harvest. The acetylation of TPI1 was analyzed by Western blotting. (H) Graphical analysis of protein acetylation by tandem-mass-tag (TMT)-based mass spectrometry of TPI1. PEP, maximal posterior error probability for peptides; PSM, number of spectra that the protein matched. (I) 293T cells were transfected with an empty vector (EV), TPI1-HA, or the TPI1-HA mutation construct as indicated. Cell lysates were immunoprecipitated with an anti-HA antibody and analyzed by Western blotting. (J) 293T cells were transfected with TPI1-HA (wild type [WT]) or the TPI1-HA mutant (K69R) construct as indicated, and the cells were stimulated with 5 ng/ml TGF-β1 as indicated for 4 h before harvesting. Cell lysates were immunoprecipitated with an anti-HA antibody and analyzed by Western blotting. (K and L) Western blot analysis of WCLs derived from 293T cells transfected with wild-type TPI1 (WT), K69R-TPI1 mutant (K69R), or K69Q-TPI1 mutant (K69Q) constructs as indicated. Cells were pretreated with 100 μg/ml cycloheximide (CHX) for the indicated times. The half-life of TPI1 was determined by the ImageJ software. F-6-P, fructose-6-phosphate; F-1,6-P, fructose-1,6-bisphosphate; G-3-P, glyceraldehyde-3-phosphatedehydrogenase; 1,3-BPG, 1,3-bisphosphoglycerate; 2-PG, 2-phosphoglyceric acid; PEP, phosphoenolpyruvate; Acetyl-CoA, acetyl coenzyme A; ATP, adenosine triphosphate; GTP, guanosine triphosphate; IgG, immunoglobulin G; AcK, acetylation of lysine; IP, immunoprecipitation; IB, immunoblotting.

Previous studies have demonstrated that the glycolytic pathway is significantly activated in pulmonary fibrosis. Our results revealed that TGF-β1-induced fibrosis were inhibited by the glycolysis inhibitor 2-deoxyglucose (Fig. 3A and B). As a critical glycolytic enzyme, TPI1 exhibited a strong correlation with disease progression, suggesting its regulatory role in fibrotic remodeling through modulation of glycolytic flux [15]. We found that TPI1 knockdown significantly attenuated TGF-β1-induced extracellular acidification rate (ECAR) elevation during fibrotic progression (Fig. 3C and D), while ECAR increased when TPI1 was overexpressed (Fig. S1H and I). This suggests that TPI1 may participate in the regulation of IPF progression through glycolysis.

In order to investigate the glycolysis-related mechanism of TPI1, we performed metabolomics analysis and discovered that the levels of acetyl coenzyme A (acetyl-CoA) were significantly elevated when TPI1 was overexpressed (Fig. 3E and F). Acetyl-CoA is a core molecule in energy metabolism and a direct donor of protein acetylation modifications. Studies have shown that enzymes in the glycolytic pathway, including pyruvate kinase isozyme type M2, enolase 1, enolase 2, and lactate dehydrogenase A, undergo acetylation and affect cellular glycolysis in various ways [28–31]. However, whether TPI1 undergoes acetylation modification remains unclear.

Intriguingly, in a TGF-β1-induced cellular model of fibroblast-to-myofibroblast transformation, we also detected multiple potential glycolytic regulatory enzymes (including TPI1) that undergo acetylation modifications via acetylation proteomics. Immunoprecipitation results showed that TGF-β1 can significantly inhibit the acetylation of TPI1 (Fig. 3G). Mass spectrometry data indicate 5 potential acetylation modification sites (K69, K149, K175, K188, and K219) of TPI1 (Fig. 3H and Fig. S2A). To determine the key lysine residue involved, we generated mutant plasmids for each of the 5 lysine sites (K to R) and conducted immunoprecipitation experiments. We found that mutating K69 led to a marked reduction in TPI1 acetylation (Fig. 3I). Evolutionary conservation analysis revealed that the K69 site of TPI1 protein was conserved across different species (Fig. S2B). Furthermore, the acetylation of the K69R mutant did not change in response to TGF-β1 stimulation (Fig. 3J).

To explore the effect of acetylation on TPI1 function, we transfected cells with K69Q acetyl-mimetic and K69R acetylation-deficient mutants. Enzyme activity tests show that both KR and KQ mutants had no significant effect on TPI1 enzyme activity (Fig. S2C). Cycloheximide (CHX) chase experiments revealed that the K69Q mutation decreased TPI1’s half-life, while the K69R mutation prolonged it (Fig. 3K and L). These findings collectively indicate that acetylation at K69 destabilizes TPI1 by accelerating its degradation.

### p300 and HDAC11 mediate the acetylation of TPI1

**Fig. 4.**
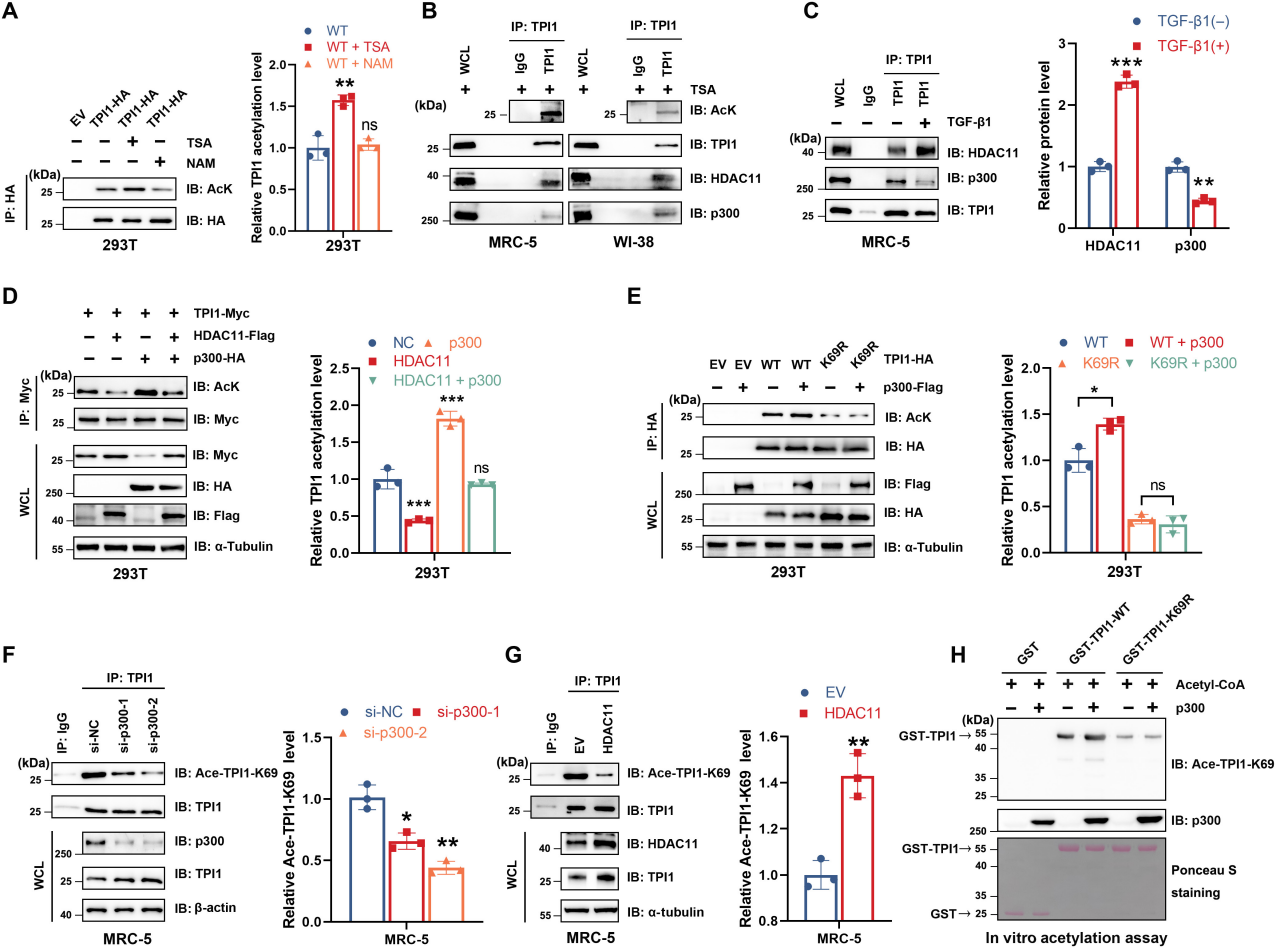
p300 and histone deacetylase 11 (HDAC11) mediate the acetylation of TPI1. (A) Western blot analysis of anti-HA immunoprecipitates derived from 293T cells transfected with EV and TPI1-HA as indicated constructs. Cells were pretreated with trichostatin A (TSA; 2 μM) or nicotinamide (NAM; 10 mM) for 1 h before harvest. (B) Western blot analysis of WCLs and anti-TPI1 immunoprecipitates derived from MRC-5 cells and WI-38 cells. Cells were pretreated with TSA (2 μM) for 1 h before harvest. The binding of TPI1 to HDAC11 or p300 was determined by immunoblotting. (C) Western blot analysis of WCLs and anti-TPI1 immunoprecipitates derived from 293T cells pretreated with 5 ng/ml TGF-β1 as indicated for 4 h before the cells were harvested. The binding of TPI1 to HDAC11 or p300 was determined by immunoblotting. (D) Western blot analysis of WCL and anti-Myc immunoprecipitates derived from 293T cells transfected with TPI1-Myc, HDAC11-Flag, and p300-HA as indicated. (E) Western blot analysis of WCL and anti-HA IPs derived from 293T cells transfected with EV, wild-type TPI1 (WT), or the K69R-TPI1 mutant (K69R) construct as indicated. (F and G) Western blot analysis of WCLs and anti-TPI1 immunoprecipitates derived from MRC-5 cells transfected with small interfering RNAs (siRNAs) targeting p300 (F) or HDAC11-Flag (G). (H) Bacteria-purified recombinant GST-TPI1-WT and GST-TPI1-K69R mutants were incubated with the recombinant proteins p300-Flag and acetyl-CoA purified from 293T cells in vitro in acetylation assay buffer. Then, IB analysis of the incubated solutions was performed with the Ace-TPI1-K69 antibody. *P < 0.05; **P < 0.01; ***P < 0.001. GST, glutathione *S*-transferase.

To identify the acetyltransferase responsible for TPI1 acetylation, we individually transfected 5 acetyltransferases, p300/CBP-associated factor (PCAF), general control of amino acid synthesis protein 5-like 2 (GCN5), CREB-binding protein (CBP), E1A binding protein P300 (p300), and Tat-interacting protein 60 kDa (TIP60α), into 293T cells, followed by co-immunoprecipitation (Co-IP) assays. We found that p300 could bind to TPI1 and significantly increase TPI1 acetylation (Fig. S3A). We then tested whether the acetylation level of TPI1 changes in response to the class I and II histone deacetylase (HDAC) inhibitor trichostatin A (TSA) or the HDAC class III inhibitor nicotinamide (NAM). The acetylation level of TPI1 was increased by TSA but not NAM (Fig. 4A), suggesting that TPI1 is deacetylated by an HDAC family deacetylase. We transiently expressed 11 human HDAC proteins in combination with TPI1 and determined their interaction. Among the tested HDACs, only HDAC11 was found to interact with and deacetylate TPI1 (Fig. S3B). Knockdown of HDAC11 abolished the effect of TGF-β on reducing TPI1 acetylation (Fig. S3C), suggesting that TGF-β mediates this reduction via HDAC11. Consistent with this observation, protein interactions between endogenous TPI1, p300, and HDAC11 were detected in MRC-5 and WI-38 cells (Fig. 4B). Immunofluorescence analysis further showed that TPI1 co-localized with p300 and HDAC11 in the nucleus (Fig. S3D), which provided a spatial basis for their interaction. Given the TGF-induced deacetylation of TPI1, we further examined the binding of TPI1 to p300 and HDAC11 under TGF-β1 stimulation. We found that the interaction between HDAC11 and TPI1 was enhanced by TGF-β1, while the interaction between p300 and TPI1 was correspondingly weakened (Fig. 4C), indicating that TGF-β1 reduces TPI1 acetylation by enhancing the interaction between TPI1 and HDAC11.

To further assess whether p300 and HDAC11 could modulate the acetylation of TPI1, rescue assays were performed. The results showed that the acetylation level of TPI1 was markedly influenced by the transfection of HDAC11 and p300, and HDAC11 attenuated the p300-mediated increase in TPI1 acetylation (Fig. 4D). Additionally, p300 failed to increase TPI1-K69R mutant acetylation (Fig. 4E). To exclude p300 effects and focus on TPI1^K69^ acetylation, we constructed an antibody specific for TPI1^K69^ acetylation (Fig. S3E). Next, we measured the change in TPI1 acetylation in cells after p300 knockdown. Using an Ace-TPI1-K69-specific antibody, we determined that TPI1^K69^ acetylation decreased following p300 knockdown in MRC-5 cells (Fig. 4F). Conversely, HDAC11 overexpression decreased the acetylation level of endogenous TPI1 in MRC-5 cells (Fig. 4G). To determine whether K69 acetylation in TPI1 is mediated by p300, we conducted an in vitro acetylation assay using purified TPI1 proteins, which corroborated our previous in vivo observations (Fig. 4H). Together, these results demonstrate that TPI1-K69 is acetylated by p300 and deacetylated by HDAC11.

### Deacetylation enhances TPI1 protein stability by attenuating K48-linked polyubiquitination

**Fig. 5.**
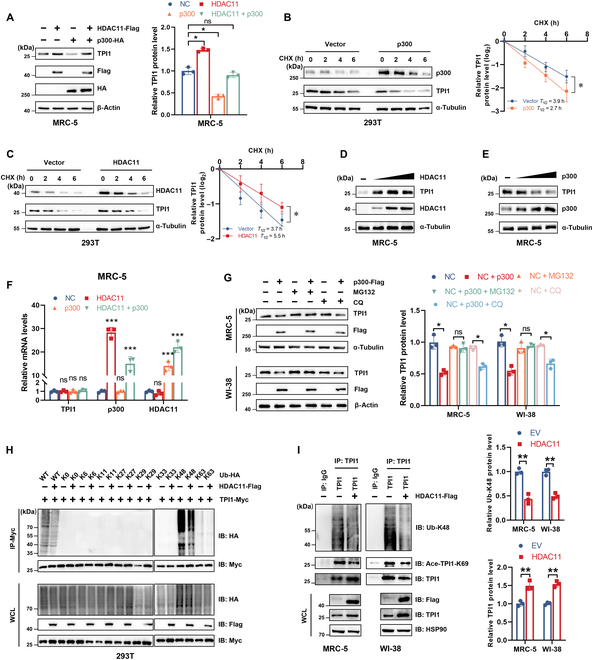
Deacetylation enhances TPI1 protein stability by attenuating K48-linked polyubiquitination. (A) Western blot analysis of TPI1 protein expression in MRC-5 cells transfected with p300-HA and HDAC11-Flag as indicated. (B and C) Western blot analysis of WCLs derived from 293T cells transfected with p300-Flag (B) and HDAC11-Flag (C) as indicated. Cells were pretreated with 100 μg/ml CHX for the indicated times. The half-life of TPI1 was determined by the ImageJ software. (D and E) Western blot analysis of WCLs derived from MRC-5 cells transfected with increasing doses (0, 1, 2, or 4 μg) of HDAC11-Flag (D) and p300-Flag (E). (F) Real-time quantitative reverse transcription polymerase chain reaction (qRT-PCR) analysis of TPI1 mRNA expression in MRC-5 cells transfected with p300-HA and HDAC11-Flag as indicated. (G) Western blot analysis of WCLs derived from MRC-5 cells (top panel) and WI-38 cells (bottom panel) transfected with p300-Flag. Cells were pretreated with MG132 (5 μM) or chloroquine (CQ; 50 μM) for 16 h before harvest. α-Tubulin and β-actin served as internal controls. (H) Western blot analysis of WCLs and anti-Myc IPs derived from 293T cells transfected with TPI1-Myc, the indicated HDAC11-Flag, WT-Ub-HA, and mutant-Ub-HA (K0, K6, K11, K27, K29, K33, K48, and K63) constructs. (I) Western blot analysis of WCLs and anti-TPI1 immunoprecipitates derived from MRC-5 cells (left panel) and WI-38 cells (right panel) transfected with the indicated HDAC11-Flag constructs. Immunoprecipitates were immunoblotted with antibodies against TPI1, Ub-K48, and Ace-TPI1-K69. WCLs were immunoblotted with antibodies against Flag, TPI1, and HSP90. HSP90 served as an internal control. *P < 0.05; **P < 0.01; ***P < 0.001.

To further explore how acetylation modification regulates the expression of TPI1, we first transfected cells with p300 and HDAC11 plasmids separately or in combination with TPI1 plasmid. The protein level of TPI1 was markedly increased by HDAC11, whereas p300 significantly decreased TPI1 expression. Moreover, HDAC11 attenuated p300-mediated accumulation of TPI1, suggesting that acetylation negatively regulated TPI1 protein levels (Fig. 5A). The half-life of endogenous TPI1 after treatment with CHX was decreased by the overexpression of p300 (Fig. 5B), while HDAC11 overexpression prolonged it (Fig. 5C). Consistently, p300- and HDAC11-triggered changes in TPI1 expression were dose dependent in MRC-5 cells (Fig. 5D and E). The above data suggest that the deacetylation of TPI1 enhances its protein stability.

Meanwhile, whether p300 and HDAC11 were overexpressed separately or in combination, TPI1 messenger RNA expression was not altered (Fig. 5F). Moreover, we found that the proteasomal inhibitor MG132 blocked the p300-induced decrease in endogenous TPI1 protein levels, but lysosomal inhibitor treatment had no effect on the abundance of TPI1 (Fig. 5G). Thus, we co-transfected wild-type (WT) or site-specific mutant Ub plasmid with HDAC11 and /or TPI1 plasmid into 293T cells. The Co-IP experiment showed that HDAC11 inhibited K48-linked TPI1 polyubiquitination (Fig. 5H). Consistent with these findings, we further confirmed that the K48-linked polyubiquitination of endogenous TPI1 was decreased in HDAC11-overexpressing MRC-5 and WI-38 cells (Fig. 5I). These results collectively showed that deacetylation enhances TPI1 protein stability by attenuating K48-linked polyubiquitination.

### Deacetylation of TPI1 promotes the progression of fibrosis

**Fig. 6.**
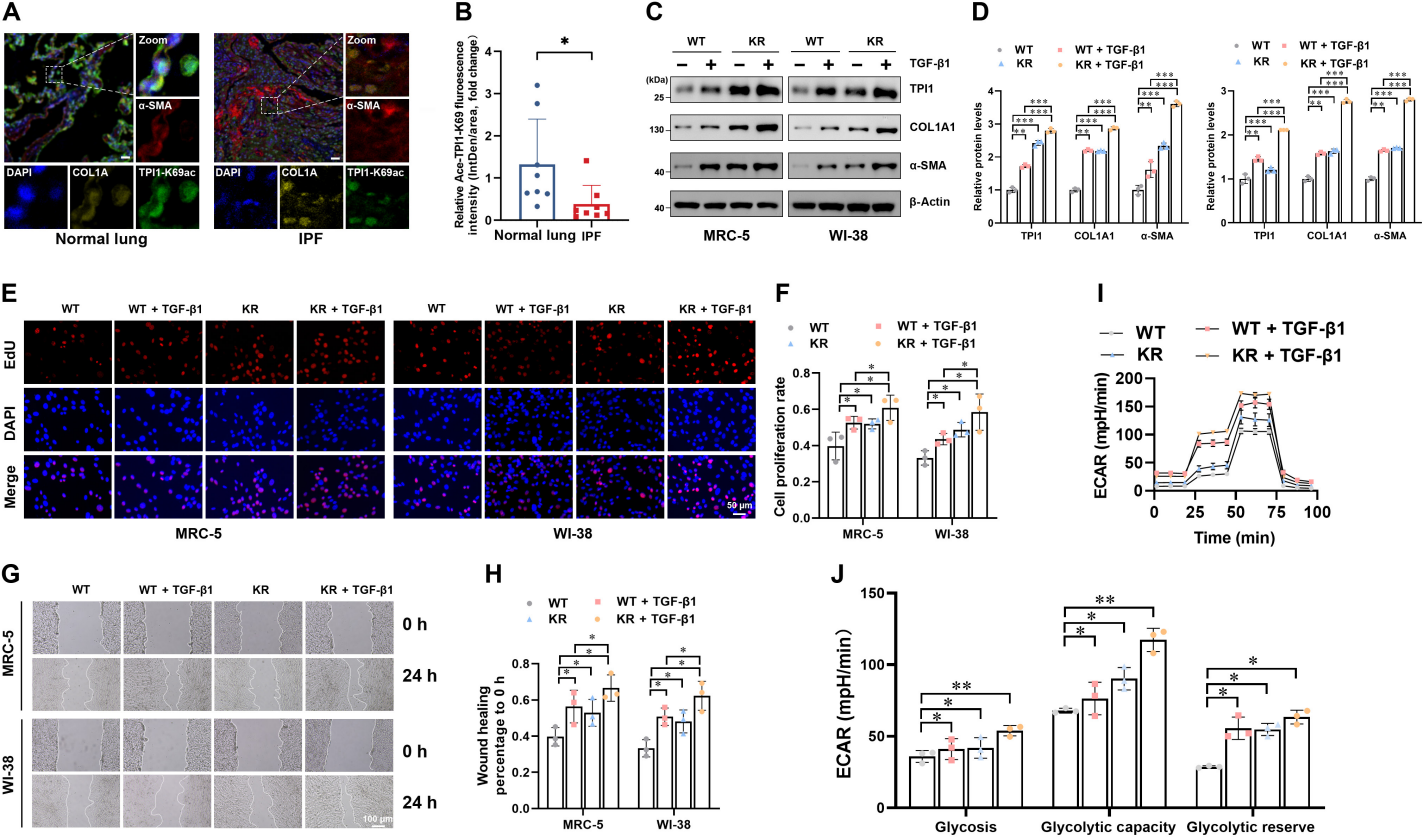
Deacetylation of TPI1 promotes fibrosis progression. (A and B) Left panel: Slides from normal lung and IPF tissues were subjected to multi-IF staining. TPI1-K69ac fluorescence (green), α-SMA fluorescence (red), COL1A1 fluorescence (yellow), and nuclear fluorescence (blue) are shown. Right panel: Quantitative analysis of the fluorescence intensity of TPI1-K69 acetylation. The scale bar represents 100 μm; *n* = 3 biological replicates for each analysis. *P < 0.05. (C and D) Western blot analysis of TPI1, α-SMA, and COL1A1 expression in WT-TPI1- and K69R-TPI1-expressing MRC-5 cells and WI-38 cells pretreated with 5 ng/ml TGF-β1 for 24 h; β-actin served as an internal control. **P < 0.01; ***P < 0.001. (E and F) EdU assays were performed in WT or K69R-TPI1-expressing MRC-5 and WI-38 cells pretreated with 5 ng/ml TGF-β1 for 24 h. See also Fig. 2C. *P < 0.05. (G and H) Wound healing assays were performed in WT or K69R-TPI1-expressing MRC-5 or WI-38 cells pretreated with 5 ng/ml TGF-β1 for 24 h. See also Fig. 2F. *P < 0.05. (I and J) Real-time ECARs were generated in WT or K69R-TPI1-expressing MRC-5 cells pretreated with 5 ng/ml TGF-β1 for 24 h. See also Fig. 3C. *P < 0.05; **P < 0.01.

To investigate the role of TPI1^K69^ acetylation in IPF progression, we collected lung tissues of IPF patients and performed multiplex immunofluorescence analysis with anti-Ace-TPI1-K69. The results revealed that TPI1^K69^ acetylation was down-regulated in IPF (Fig. 6A and B). Next, we investigated whether TPI1^K69^ deacetylation maintained TPI1 function. To this end, TPI1 was knocked down with a specific short hairpin RNA (shRNA) targeting the 5′-untranslated region of TPI1; then, TPI1-WT and TPI1-K69R overexpression plasmids were reintroduced (Fig. S4A and B). Endogenous TPI1 was successfully depleted and replaced with TPI1-WT or TPI1-K69R, as verified by Sanger sequencing (Fig. S4C). Moreover, we pretreated cells with TGF-β1 to induce fibrosis; the expression of the fibrosis markers COL1A1 and α-SMA was increased in TPI1-K69R cells compared with those in TPI1-WT cells (Fig. 6C and D). Additionally, we found that the TPI1 K69R mutant had a greater ability to promote IPF progression than did TPI1-WT in cell proliferation (Fig. 6E and F) and migration (Fig. 6G and H). Furthermore, compared with WT-TPI1, MRC-5 and WI-38 cells overexpressing TPI1-K69R exhibited increased glycolysis, glycolytic capacity, and glycolytic reserve (Fig. 6I and J). Collectively, these data demonstrate that the deacetylation of TPI1^K69^ promotes the progression of fibrosis.

### CPP-induced TPI1 K69 acetylation inhibits IPF

**Fig. 7.**
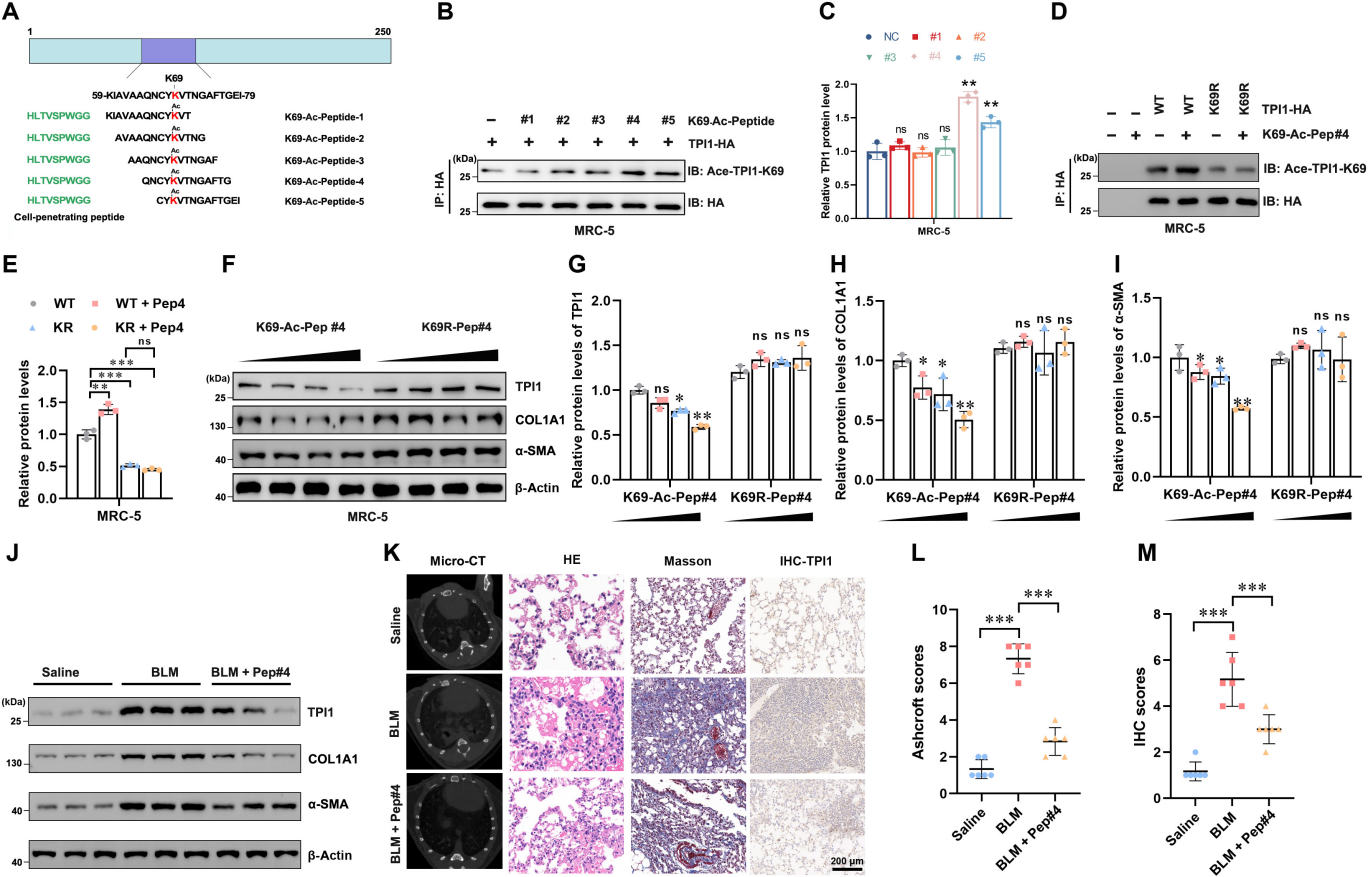
Cell penetrating peptide (CPP)-induced TPI1 K69 acetylation inhibits IPF. (A) Schematic representation of the TPI1 protein structure highlighting the K69 acetylation site and the sequences of synthesized K69-acetylated peptides (#1 to #5); Green: CPPs; red: site for TPI1 acetylation. (B and C) Immunoblot analysis of TPI1 acetylation in MRC-5 cells treated with K69-acetylated peptides (#1 to #5). HA-tagged TPI1 was immunoprecipitated, and K69 acetylation was detected. **P < 0.01; ns, no significance. (D and E) Effect of empty vector, TPI1-WT, and K69R mutation acetylation after treatment with K69-Ac-Pep#4 in MRC-5 cells. HA-tagged TPI1 was immunoprecipitated, and K69 acetylation was detected. **P < 0.01; ***P < 0.001; ns, no significance. (F to I) Dose-dependent effect (0, 1, 5, and 10 mM) of K69-Ac-Pep#4 and K69R-Pep#4 in MRC-5 cells. Western blot analysis of TPI1, α-SMA, and COL1A1 protein expression. *P < 0.05; **P < 0.01; ns, no significance. (J) Western blot analysis of TPI1, α-SMA, and COL1A1 expression in the lung tissue of mice; β-actin served as an internal control. (K to M) Left panel: representative images of micro-CT, HE staining, Masson staining, and IHC staining in mice (K). The mice were divided into groups as indicated; the minimal number of mice was used (*n* = 6 per group). The scale bar represents 200 μm. Right panel: analysis of Ashcroft scores of collagen expression (L) and IHC scores of TPI1 expression (M) in mouse lungs. ***P < 0.001.

Given the pivotal role of acetylation modification at the K69 site of TPI1 in regulating fibrosis, we synthesized multi-cell-penetrating peptides that specifically inhibit the acetylation modification at the K69 site of TPI1 and tested their therapeutic effect on IPF (Fig. 7A). MRC-5 cells were treated with CPPs, and immunoprecipitation assays revealed that K69-Ac-Peptide#4 significantly increased the acetylation of TPI1 at the K69 site compared to other peptides (Fig. 7B and C).

Further analysis showed that treatment with K69-Ac-Peptide#4 led to a marked increase in K69 acetylation in WT TPI1, but not in the K69R mutant, confirming the specificity of the peptide (Fig. 7D and E). To assess the effect of K69-Ac-Peptide#4 on TPI1 stability, MRC-5 cells were treated with increasing concentrations of the peptide. Western blot analysis demonstrated a dose-dependent decrease in TPI1, COL1A1, and α-SMA protein levels with K69-Ac-Peptide#4 treatment. In contrast, the K69R mutant did not show a similar degradation pattern (Fig. 7F to I), indicating that K69-Ac-Peptide#4 promotes TPI1 degradation by enhancing K69 acetylation. In vivo validation further demonstrated that K69-Ac-Peptide#4 administration significantly reduced TPI1 expression, attenuated pulmonary fibrotic lesions, and decreased pulmonary collagen deposition in an IPF model (Fig. 7J to M). These results suggest that inhibiting TPI1 acetylation modification through the CPP approach may be a viable strategy for treating IPF.

## Discussion

In this study, we found that TPI1 expression was elevated in IPF and TGF-β1 increased TPI1 protein expression by reducing its acetylation. Specifically, HDAC11 mediated deacetylation at K69, which enhanced its protein stability by attenuating K48-linked polyubiquitination. Furthermore, our study suggests that CPPs targeting TPI1-K69ac may offer a therapeutic strategy for IPF by inhibiting its acetylation.

TPI1 plays a pivotal role in fibrotic progression, rendering the targeted regulation of its expression a critical research focus. We used a novel strategy to explore the molecular regulatory mechanisms of TPI1 by investigating the potential regulatory factors involved in metabolic reprogramming. Notably, our findings demonstrate that lysine acetylation serves as a crucial posttranslational modification governing TPI1 protein stability. Acetylation plays key roles in protein stability, subcellular localization, and protein–protein interactions [32–34]. Zhao et al.’s studies showed that most metabolic enzymes can be acetylated through acetylation modification omics [24,35–37]. Here, we found that the glycolytic enzyme TPI1 was acetylated by p300 or deacetylated by HDAC11. This confirms the strong correlation between metabolic enzymes and acetylation modifications.

Acetylation modifications are widely involved in the progression of various diseases [38,39]. For example, Ding et al. [40] demonstrated that C/EBPβ acetylation may play a central role in pulmonary fibrosis. Zhang et al. [41] found that regulating the level of H4K16Ac can reduce the expression of pro-fibrotic genes and alleviate the degree of lung fibrosis in aged mice. The above research prompted the development of synthesized materials to assess acetylation in pulmonary fibrosis models. Lv et al. synthesized a fluorescent probe to evaluate the role of HDAC in pulmonary fibrosis cells and mice [42–45]. Inspired by Chen et al.’s research [46], we developed a novel method using CPPs to deliver and target the acetylated peptide of TPI1. However, a limitation is the peptide’s lack of inherent target specificity. For instance, while it effectively reduces TPI1 levels and fibrotic activity, its nonspecificity may result in off-target effects by altering the acetylation of other proteins. This could complicate the clinical translation of this approach. Future research should focus on enhancing the selectivity of the peptide to improve its therapeutic potential while minimizing unintended consequences.

The cross talk between acetylation and ubiquitination modifications plays a crucial role in the regulation of TPI1 stability and the progression of IPF. However, in this study, we did not find a specific lysine residue that is polyubiquitinated or detect E3 ligase-induced K48 polyubiquitination of TPI1, possibly due to the low abundance of polyubiquitinated TPI1 or the limitation of detection methods. A more detailed exploration of the mechanisms mediating this cross talk between acetylation and ubiquitination is planned for future studies.

In summary, we observed that TGF-β induces the upregulation of TPI1, which contributes to IPF development. Our findings elucidate a novel mechanism involving p300- and HDAC11-mediated TPI1 acetylation, which regulates TPI1 stability via K48-linked polyubiquitination. Hence, targeting TPI1 acetylation represents a promising and rational strategy for IPF treatment.

## Methods

### Cell lines and cell culture

The MRC-5, WI-38, and 293T cell lines were purchased and validated from Fuheng Biotechnology (Shanghai, China). The cultivation of MRC-5 and WI-38 cells was performed in 90% minimum essential medium (Gibco, no. 11095080) supplemented with the addition of 10% fetal bovine serum (FBS; ABW, no. AB-FBS0500S) and 100 units/ml penicillin and streptomycin (Fuheng, no. FH-K001T) [26]. The 293T cells were cultivated in Dulbecco’s modified Eagle medium (DMEM; Gibco, no. 11965167), which was enriched with 10% FBS (ABW, no. AB-FBS0500S), in addition to 100 units/ml penicillin and streptomycin (Fuheng, no. FH-K001T). TGF-β1 (5 ng/ml) was added at the concentrations indicated in the figures or figure legends after 12 h of 1% serum starvation. A CHX chase assay was performed by treating cells with 100 μg/ml CHX for the indicated times. Cells were usually treated with targeted CPPs at a concentration of 20 μM or the indicated concentration in CPP treatment assay.

### Human IPF and donor tissue specimens

Eight IPF patient lung tissue specimens and 8 donor lung tissue specimens were collected from Huashan Hospital (Shanghai, China). All patients were diagnosed with IPF according to international consensus guidelines. We obtained informed consent from the patients and donors. This study was approved by the Ethics Committee of Fudan University (KY2023-015).

### Plasmids

The pCMV-p300-Flag plasmid (Miaolingbio, no. P56573) was purchased from Miaolingbio. The pcDNA3.1-Flag, pcDNA3.1-Myc, pCMV-Flag, PGEX-4T2-GST, pCDH-HA, psPAX, and pMD2.G vector plasmids were kind gifts from Zhang-HT’s laboratory (Soochow University, China). The remaining plasmids used in the study were synthesized by our laboratory. The plasmids were constructed as we previously described [27].

### Antibodies and reagents

The following antibodies were used in this paper: TPI1 (Proteintech, no. 10713-1-AP), α-SMA (ABclonal, no. A1399), COL1A1 (ABclonal, no. A1352), α-tubulin (ABclonal, no AC007), β-actin (ABclonal, no. AC038), HSP90 (ABclonal, no. A0365), HA (CST, no. 3724s), Flag (CST, no. 14793s), Myc (CST, no. 2276S), p300 (Santa Cruz, no. sc-32244), HDAC11 (ABclonal, no. A6140), pan acetyl-lysine (ABclonal, no. A1525), rabbit anti-immunoglobulin G (anti-IgG) (CST, no. 3900), mouse anti-IgG (CST, no. 5415S), Ace-K69-TPI1 (Cohesion, no. K2218), horseradish peroxidase (HRP)-conjugated goat anti-rabbit (ABclonal, no. AS029), and HRP-conjugated goat anti-mouse (ABclonal, no. AS014).

The following reagents were used in this study: TSA (Sigma, no. T8552), NAM (Sigma, no. N0636), CHX (MCE, no. HY-12320), acetyl-CoA trisodium (MCE, no. HY-113596), MG132 (MCE, no. HY-13259), chloroquine (MCE, no. HY-17589A), and BLM hydrochloride (MCE, no. HY-17565A).

### Real-time quantitative reverse transcription polymerase chain reaction

RNA was isolated from cells using Cell Total RNA Isolation Kit (Vazyme, no. RC113-01). RNA quality was tested using a NanoDrop 2000 spectrophotometer with acceptance criteria. Complementary DNA synthesis was conducted using 500 to 1,000 ng of total RNA with HiScript III RT Super Mix (ABclonal, no. RK20432). Quantitative polymerase chain reaction (qPCR) was conducted on a Roche LightCycler 96 system (Roche Diagnostics) using ChamQ SYBR qPCR Master Mix (Vazyme, no. Q711-02). Each 20-μl reaction contained 10 μl of SYBR Green mix, 0.4 μM forward/reverse primers (sequences in Table S1), 2 μl of complementary DNA template, and nuclease-free water. The thermal cycling parameters were as follows: 95 °C for 30 s, initial denaturation; followed by 40 cycles of 95 °C for 10 s and 60 °C for 30 s. Melting curve analysis was performed by gradually increasing the temperature from 65 to 95 °C at increments of 0.5 °C per step. All samples were run in technical triplicate, and β-actin (ACTB) was used as the reference gene.

### Construction of stable shRNA/siRNA-transfected cell lines

#### shRNA lentiviral system

shRNAs targeting TPI1 (sequences in Table S1) were cloned into the pLKO.1-puro vector (Tsingkebio, Shanghai). Lentiviral particles were produced in HEK293T cells by co-transfecting psPAX2 and pMD2.G packaging plasmids (Addgene no. 12260 and no. 12259) using Lipofectamine 3000. The viral supernatant was harvested at 48 and 72 h posttransfection, filtered through a 0.45-μm membrane, and concentrated via ultracentrifugation at 50,000×g at 4 °C for 2 h. Target cells were transduced with viral particles at a multiplicity of infection of 5 in the presence of 8 μg/ml polybrene for 24 h. The cells were then cultured for 7 d in a medium containing 2 μg/ml puromycin. Knockdown efficiency was validated by real-time quantitative reverse transcription polymerase chain reaction and Western blotting.

#### siRNA transfection

Small interfering RNA (siRNA) duplexes targeting p300 (GenePharma, Shanghai) were transfected at a 50 nM final concentration using jetPRIME (Polyplus, no. 101000001). Cells were seeded at 70% confluency in an antibiotic-free medium 24 h prior to transfection. Complexes were prepared by mixing siRNA with jetPRIME buffer at a 1:2 v/v ratio and incubated for 10 min at room temperature (RT). The medium was replaced 6 h posttransfection, and cells were harvested after 48 h for analysis.

### Co-IP and Western blotting

Cells were lysed in ice-cold IP buffer (50 mM Tris–HCl pH 7.5, 150 mM NaCl, 10% glycerol, 0.5% NP-40, and protease and phosphatase inhibitor cocktail) for 30 min. Lysates were clarified by centrifugation at 14,000×g for 15 min at 4 °C, and the protein concentration was determined via bicinchoninic acid assay. For Co-IP, 500 μg of lysate was incubated overnight at 4 °C with 20 μl of anti-Flag magnetic beads (Sigma, no. M8823) or 2 μg of anti-TPI1 antibody coupled to Protein A/G beads (Vazyme, no. PB101-01). The beads were washed 3 times with IP buffer (10 min per wash at RT) and eluted in 5× loading buffer at 95 °C for 5 min.

Western blotting was performed as described. Proteins were resolved on 10% sodium dodecyl sulfate–polyacrylamide gel electrophoresis (SDS-PAGE) gels, transferred to polyvinylidene difluoride or nitrocellulose membranes, and blocked with 5% nonfat milk in ‌Tris-buffered saline with Tween 20 for 1 h. Primary antibodies anti-TPI1 (1:1,000), anti-acetyl-lysine (1:2,000), and anti-β-actin (1:5,000) were incubated overnight at 4 °C. HRP-conjugated secondary antibodies were incubated for 1.5 h at RT. Then, membranes were detected using enhanced chemiluminescence on a ChemiDoc system.

### Recombinant protein synthesis

#### p300-Flag purification

HEK293T cells at 80% confluency in DMEM + 10% FBS were transfected with 2 μg of pcDNA3.1-p300-Flag using Lipofectamine 3000. Cells were harvested 48 h posttransfection in radioimmunoprecipitation assay buffer, and lysates were incubated with anti-Flag M2 affinity gel (Yeasen, no. 20584ES03) for 4 h at 4 °C. Beads were washed 3 times with Tris-buffered saline and eluted with 200 μg/ml 3× Flag peptide for 30 min at RT.

#### GST-TPI1 purification

BL21 *Escherichia coli* transformed with pGEX-4T2-TPI1 plasmids were grown in TB medium + 100 μg/ml ampicillin at 37 °C to OD_600_ = 0.6. Protein expression was induced with 0.1 mM isopropyl-β-D-thiogalactopyranoside‌ at 20 °C for 16 h. Cells were lysed by sonication for 5 cycles: 30 s on/30 s off. Cleared lysates were incubated with glutathione-Sepharose 4B (Sangon, no. C600911) for 1 h at 4 °C. Beads were washed with 10 column volumes of phosphate-buffered saline (PBS) and eluted with 10 mM reduced glutathione pH 8.0. Protein purity was confirmed by SDS-PAGE and Coomassie staining.

### In vitro acetylation assay

Recombinant GST-TPI1 or GST-TPI1-K69R (2 μg) was incubated with 1 μg of p300-Flag in acetylation buffer (50 mM Tris–HCl pH 7.5, 50 mM KCl, 5% glycerol, 100 nM TSA, 1 mM acetyl-CoA, and 1 mM dithiothreitol) for 1 h at 30 °C. Reactions were terminated by adding 5× loading buffer, and acetylation was analyzed by immunoblotting with anti-acetyl-lysine antibody. Glutathione *S*-transferase (GST)-tagged proteins were visualized via Ponceau S staining.

### Multiplex immunofluorescence

Lung sections from 8 patients diagnosed with IPF and 8 controls were deparaffinized in xylene and then rehydrated through graded ethanol. Antigen retrieval was conducted in a sodium citrate buffer (10 mM, pH 6.0) at a temperature of 95 °C for a duration of 20 min. Sections were blocked with 3% bovine serum albumin and 0.3% Triton X-100 for 1 h, after which they were incubated overnight at 4 °C with primary antibodies. These were anti-TPI1 (1:100), anti-COL1A1 (1:100), and anti-α-SMA (1:100). Tyramide signal amplification (Absin, no. abs50012) was performed per the manufacturer’s protocol, with 1 μg/ml 4′,6-diamidino-2-phenylindole for 5 min for nuclear staining. Slides were mounted with ProLong Diamond Antifade (Invitrogen, no. P36961) and imaged on a Zeiss LSM710 confocal microscope (×63 oil objective, z-stack 0.5-μm intervals). Image analysis was performed using ZEN 3.0 Zeiss and Fiji/ImageJ with threshold-based quantification.

### Seahorse XFe24 extracellular flux analysis

A total of 1 × 10^5^ MRC-5 or WI-38 cells were seeded per well in Seahorse XF-24 cell culture microplates (Agilent, no. 100777-004) and cultured overnight in complete DMEM. Plates were equilibrated for 1 h in a non-CO_2_ incubator at 37 °C prior to assay. The compounds were loaded into the injection ports in the following manner: Port A was connected to a solution of 10 mM glucose, Port B was connected to a solution of 1 μM oligomycin, and Port C was connected to a solution of 50 mM 2-deoxyglucose. Three baseline measurements were recorded before sequential compound injections. ECAR (mpH/min) was monitored every 5 min for 90 min using the Seahorse XFe24 analyzer. Data were normalized to total protein content via bicinchoninic acid assay and analyzed using Seahorse Wave Controller 2.6 (Agilent).

### Cell proliferation assay

Cells were seeded into 12-well plates at 5 × 10^4^ cells/well. At 70% confluence, 50 μM EdU (Beyotime, C0078S) was added to complete medium for 4 h at 37 °C/5% CO_2_. Cells were fixed with 4% paraformaldehyde for 15 min, permeabilized with 0.3% Triton X-100 for 20 min, and incubated with a Click-iT reaction cocktail containing Alexa Fluor 594 azide for 30 min protected from light. Nuclei were counterstained with 5 μg/ml Hoechst 3334 for 10 min. Three random fields/well were imaged using a microscope. EdU-positive cells were quantified using ImageJ.

### Wound healing assay

Confluent monolayers in 6-well plates were scratched using a 20-μl sterile pipette tip. Debris was removed by PBS washing. Images were captured at 0 and 24 h using a microscope. Wound areas were measured using ImageJ.

### Peptide synthesis

The CPP was synthesized and purified via high-performance liquid chromatography by Genescript Bio (Nanjing, China). The peptide was dissolved in sterile PBS to prepare a stock solution at a concentration of 10 mM and stored at −80 °C until use. The sequences of the CPP are listed as follows: #1, HLTVSPWGGKIAVAAQNCYK[ac]VT; #2, HLTVSPWGGAVAAQNCYK[ac]VTNG; #3, HLTVSPWGGAAQNCYK[ac]VTNG-AF; #4, HLTVSPWGGQNCYK[ac]VTNGAFTG; #5, HLTVSPWGGCYK[ac]VTNG AFTGEI; and K69R-Pep#4, HLTVSPWGGQNCYRVTNGAFTG.

### BLM-induced pulmonary fibrosis model

Female C57BL/6J mice (8 weeks old, 20 ± 2 g) were anesthetized with 50 mg/kg pentobarbital intraperitoneally. Intratracheal instillation was performed using a 24G angiocatheter with 50 μl of saline (control) or 5 mg/kg BLM (model). AAV6-shTPI1 (1 × 10^13^ vg/ml) was administered 5 d pre-BLM. Mice were euthanized at 7, 14, or 21 d postinjection. Lungs were inflation-fixed with 4% paraformaldehyde at 25 cm H_2_O pressure for 24 h, paraffin-embedded, and sectioned at 5-μm thickness. Tissues were subjected to HE staining, Masson staining, and immunohistochemical staining. The mice were randomized into groups, and there were more than 3 mice in each group. The adeno-associated virus was synthesized by WeiZhen Bio (Shandong, China). K69-Ac-Peptide#4 (5 mg/kg) was injected via the trachea once a day.

### Micro-CT imaging

Mice under 2% isoflurane anesthesia were scanned in prone position using a Hiscan XM system. The reconstruction parameters were as follows: Feldkamp algorithm, 50-μm isotropic voxels, and medium smooth kernel. Lung density was quantified in Hiscan Analysis Suite v2.3 using fixed Hounsfield unit thresholds of −200 to 0 HU for aerated lung tissue.

### Tandem-mass-tag-based acetylome profiling

Cells were lysed in 8 M urea buffer containing 1× complete protease inhibitors, 3 μM TSA, and 50 mM NAM. Proteins were reduced with 10 mM dithiothreitol for 30 min at 56 °C, alkylated with 20 mM iodoacetamide for 15 min in the dark, and digested with Trypsin Gold Promega for 16 h. Peptides were labeled with TMT11plex reagents following the manufacturer’s instructions. A labeling efficiency >98 was verified by MS3 analysis. Analysis was performed on a Q Exactive HF-X system coupled to EASY-nLC 1200, using a 120-min gradient. Data were processed with Max Quant 2.1.3 against the UniProt human database. Acetylation sites were filtered at 1% false discovery rate with a localization probability >0.75. Protein acetylation was quantitatively analyzed by tandem-mass-tag-based mass spectrometry by Jingjie PTM BioLab Co., Ltd. (Hangzhou, China).

### Triosephosphate isomerase activity assay

The assay was performed followed with a TPI1 activity assay kit (Abcam, no. ab197001). Approximately 1 × 10^6^ cells were seeded into each well of a 24-well plate, transfected with TPI1 WT and mutant plasmids, and harvested after 48 h. After washing the cells with pre-cooled PBS, they were lysed using 100 μl of Buffer 2 on ice for 10 min. The cells lysate was then centrifuged at 4 °C and 10,000×g for 5 min to collect the supernatant. After adding 50 μl of Reaction Mix, OD_450_ was measured immediately and again after incubation at 37 °C for 30 min.

### Statistical and ethical compliance

All experiments included ≥3 biological replicates. Animal studies followed ARRIVE guidelines and were approved by the Soochow University IACUC Protocol (#202311A0273). Randomization was performed using Research Randomizer. Data are presented as mean ± standard error of the mean; statistical analyses used GraphPad Prism 9.4 with analysis of variance followed by Tukey’s post hoc test with *α* = 0.05.

## Ethical Approval

For clinical samples, all patients signed an informed consent form issued by the Ethics Committee of Fudan University (approval number: KY2023-015). All mouse experiment procedures and protocols were evaluated and authorized by the Soochow University Laboratory Animal Center and the Animal Ethics Committee of Soochow University (approval number: 202311A0273).

## Data Availability

The data underlying this article will be shared on reasonable request to the corresponding authors.

## Supplementary Materials

Supplementary 1 Graphical Abstract Figs. S1 to S4 Table S1
