# Supplementary material for: HDAC11-Mediated Deacetylation of Triosephosphate Isomerase 1 Promotes Idiopathic Pulmonary Fibrosis
Source: Research (Wash D C). 2025 Oct 16;8:0953. doi: 10.34133/research.0953 (PMC12529298; doi:10.34133/research.0953)
Supplement: Supplementary 1 — Graphical Abstract Figs. S1 to S4 Table S1 [file research.0953.f1.zip › Supporting Information.docx]

**Figure S1 Overexpression of TPI1 promoted the progression of IPF**

(A-B) Overexpression of TPI1 in MRC-5 and WI-38 cells pretreated with 5 ng/ml TGF-β1 for 24 h. Western blot analyses of TPI1, α-SMA and COL1A1 expression in the indicated cells;β-actin served as an internal control.

(C-D) An EdU assay was performed to evaluate the proliferation of TPI1-overexpressing cells pretreated with 5 ng/ml TGF-β1 for 24 h. The percentage of EdU-positive cells was calculated by using ImageJ. The scale bar represents 50 μm, n=3 biological replicates for each analysis. The data are shown as the means ± SDs (n = 3). *, p < 0.05 by unpaired Student’s t test.

(F-G) A wound healing assay was performed to evaluate the migration of cells overexpressing TPI1. The extent of wound healing was recorded and quantified by using ImageJ. The scale bar represents 100 μm, n=3 biological replicates for each analysis. The data are shown as the means ± SDs (n = 3). *, p < 0.05 by unpaired Student’s t test.

(H-I) Real-time ECARs of MRC-5 cells overexpressing TPI1 according to a glycolysis stress test and semiquantitative analysis of glycolysis, glycolytic capacity and the glycolytic reserve. n = 3, error bars, means ± SDs; all analyses were performed using one-way ANOVA with Tukey’s post hoc test, *, p < 0.05. **, p < 0.01.

**Figure S2 The acetylation site of TPI1**

1. Mass spectrum of the predicted acetylation sites of the TPI1 protein (K69, K149, K176, K188, and K219).
2. Sequence alignment of the putative acetylation sites of TPI1 in various species.
3. TPI1 WT and KR, KQ mutant plasmids were transfected in MRC-5 and WI-38 cells, respectively, and changes in TPI1 enzyme activity were detected by Triose Phosphate Isomerase Activity Assay. ns, no significance.

**Figure S3 p300 and HDAC11 interaction with TPI1**

1. Western blot analysis of WCLs and anti-HA IPs derived from 293T cells transfected with EV1 (pcDNA3.1-Flag), EV2 (pCDH-HA), PCAF-Flag, GCN5-Flag, CBP-Flag, p300-Flag, TIP60α-Myc and TPI1-HA as indicated. The binding of TPI1 to acetyltransferases was determined by immunoblotting.
2. Western blot analysis of whole-cell lysates (WCLs) and anti-HA IPs derived from 293T cells transfected with EV1 (pcDNA3.1-Flag), EV2 (pCDH-HA), HDAC-Flag and TPI1-HA as indicated. The binding of TPI1 to HDACs was determined by immunoblotting.
3. Western blot analysis of whole-cell lysates (WCLs) and anti-Myc IPs derived from 293T cells transfected with TPI1-Myc and si-HDAC11. Cells were pretreated with TGF-β1 for 4 h before harvest. The acetylation of TPI1 was determined by immunoblotting.
4. MRC-5 and WI-38 cells were subjected to multi-IF staining. TPI1 fluorescence is shown in green, α-SMA fluorescence is shown in red, COL1A1 fluorescence is shown in yellow, and nuclear fluorescence is shown in blue. The scale bar represents 100 μm, n=3 biological replicates for each analysis.
5. Elisa and dot blot analyses using the indicated synthetic TPI1 peptides with or without acetylation at the K69 residue to validate the generated Ac-TPI1-K69 antibody

**Figure S4 Generation and validation of TPI1 mutant cell lines**

(A and B) qRT‒PCR and Western blot analysis of TPI1 mRNA (A) and protein (B) expression in MRC-5 and WI-38 cells. Cells were infected with lentivirus packaged with a shRNA targeting the 5’-UTR of TPI1 and a wild-type-TPI1 (WT) or K69R mutant-TPI1 (K69R) exogenous overexpression vector.

(C) Results of DNA sequencing of TPI1-WT and TPI1-K69R MRC-5 cells (top panel) and WI-38 cells (lower panel).
