# Supplementary material for: HDAC11-Mediated Deacetylation of Triosephosphate Isomerase 1 Promotes Idiopathic Pulmonary Fibrosis
Source: Research (Wash D C). 2025 Oct 16;8:0953. doi: 10.34133/research.0953 (PMC12529298; doi:10.34133/research.0953)
Supplement: Supplementary 1 — Graphical Abstract Figs. S1 to S4 Table S1 [file research.0953.f1.zip › Table1.docx]

Table1

| Primer for qRT-PCR | Sequences (5' – 3') |
| --- | --- |
| TPI1-F | CTCATCGGCACTCTGAACG |
| TPI1-R | GCGAAGTCGATATAGGCAGTAGG |
| p300-F | AGCCAAGCGGCCTAAACTC |
| p300-R | TCACCACCATTGGTTAGTCCC |
| HDAC11-F | ACCCAGACAGGAGGAACCATA |
| HDAC11-R | TGATGTCCGCATAGGCACAG |
| COL1A1-F | GAGGGCCAAGACGAAGACATC |
| COL1A1-R | CAGATCACGTCATCGCACAAC |
| ACTA2-F | AAAAGACAGCTACGTGGGTGA |
| ACTA2-R | GCCATGTTCTATCGGGTACTTC |
| ACTB-F | CATGTACGTTGCTATCCAGGC |
| ACTB-R | CTCCTTAATGTCACGCACGAT |

| shRNA | Sequences (5' – 3') |
| --- | --- |
| sh-NC | CCGGGGACAAAGTCAGTCAGGTTATCTCGAGATAACCTGACTGACTTTGTCCTTTTTG |
| sh-TPI1-1 | CCGGTGATGTGGATGGCTTCCTTGTCTCGAGACAAGGAAGCCA-TCCACATCATTTTTT |
| sh-TPI1-2 | CCGGCTCAGAGAGAAGGCATGTCTTCTCGAGAAGACATGCCTT-CTCTCTGAGTTTTTT |

| siRNA | Sequences (5' – 3') |
| --- | --- |
| si-NC | UUCUCCGAACGUGUCACGUTT |
| si-p300-1 | CAATTCCGAGACATCTTGAGATT |
| si-p300-2 | CCCGGTGAACTCTCCTATAATTT |
| si-HDAC11 | GACTCCATACTTAATCTGTTT |
