# Supplementary figures and images for: HDAC11-Mediated Deacetylation of Triosephosphate Isomerase 1 Promotes Idiopathic Pulmonary Fibrosis
Source: Research (Wash D C). 2025 Oct 16;8:0953. doi: 10.34133/research.0953 (PMC12529298; doi:10.34133/research.0953)

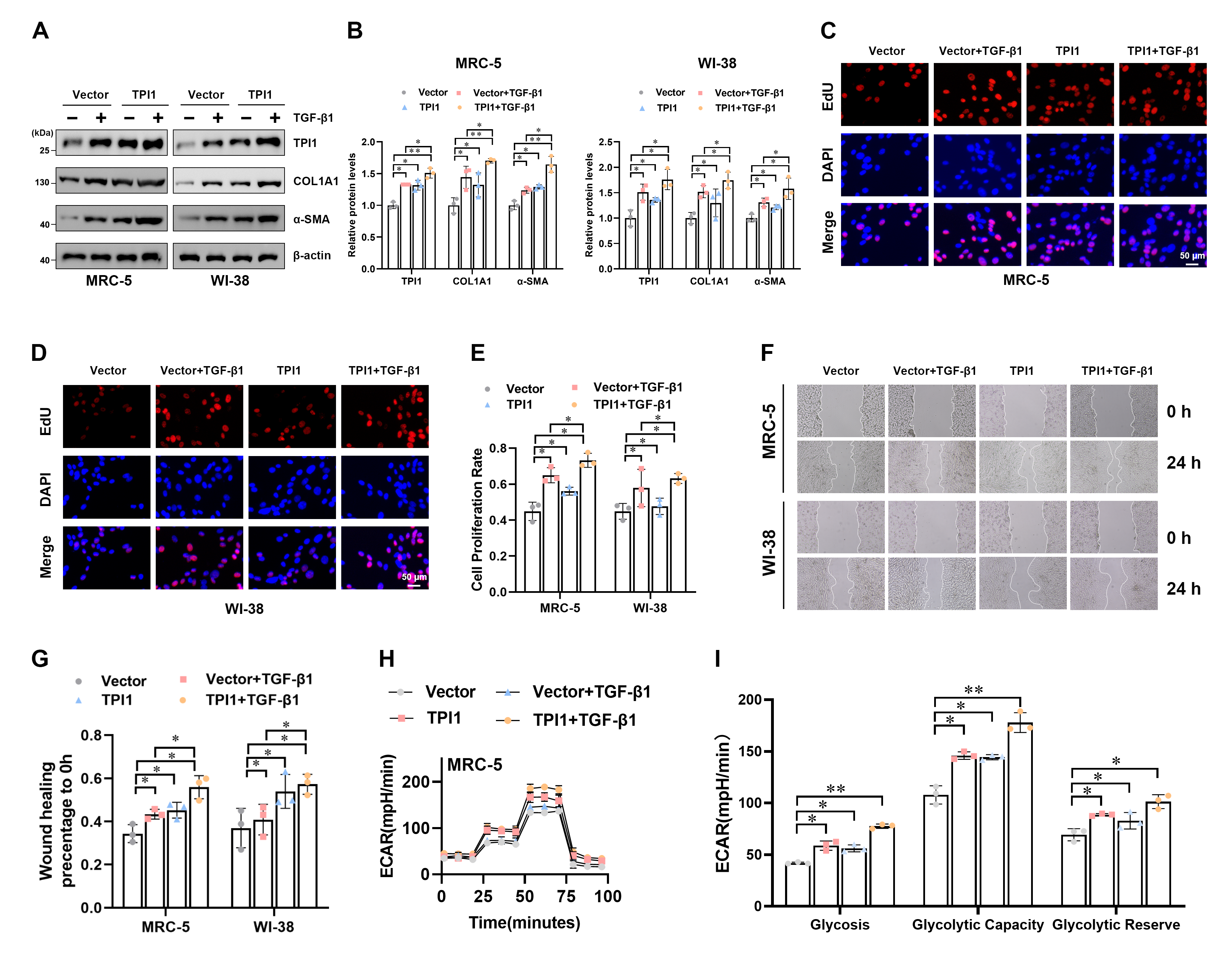

Supplement: Supplementary 1 — Graphical Abstract Figs. S1 to S4 Table S1 [file research.0953.f1.zip › FigureS1.tif]

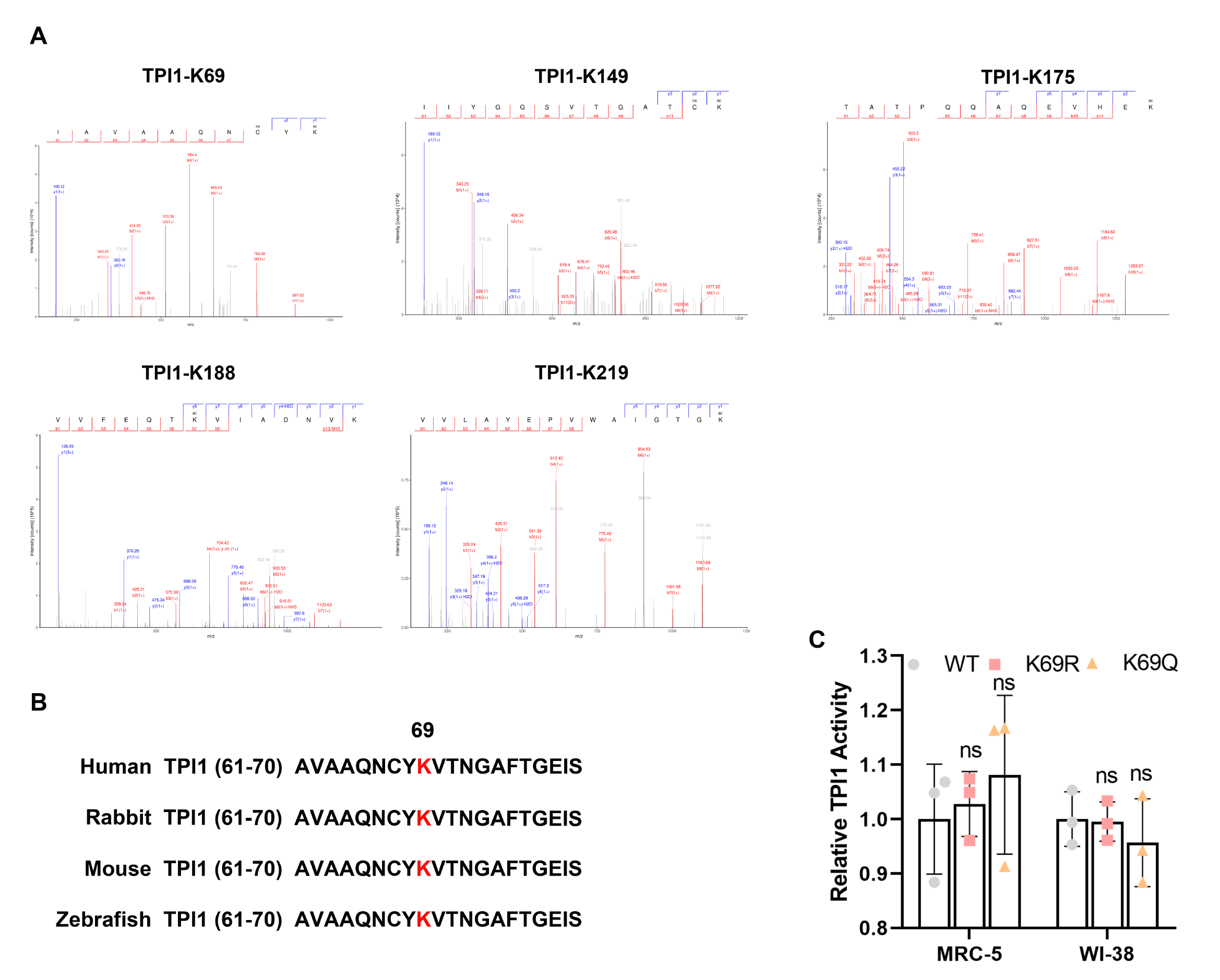

Supplement: Supplementary 1 — Graphical Abstract Figs. S1 to S4 Table S1 [file research.0953.f1.zip › FigureS2.tif]

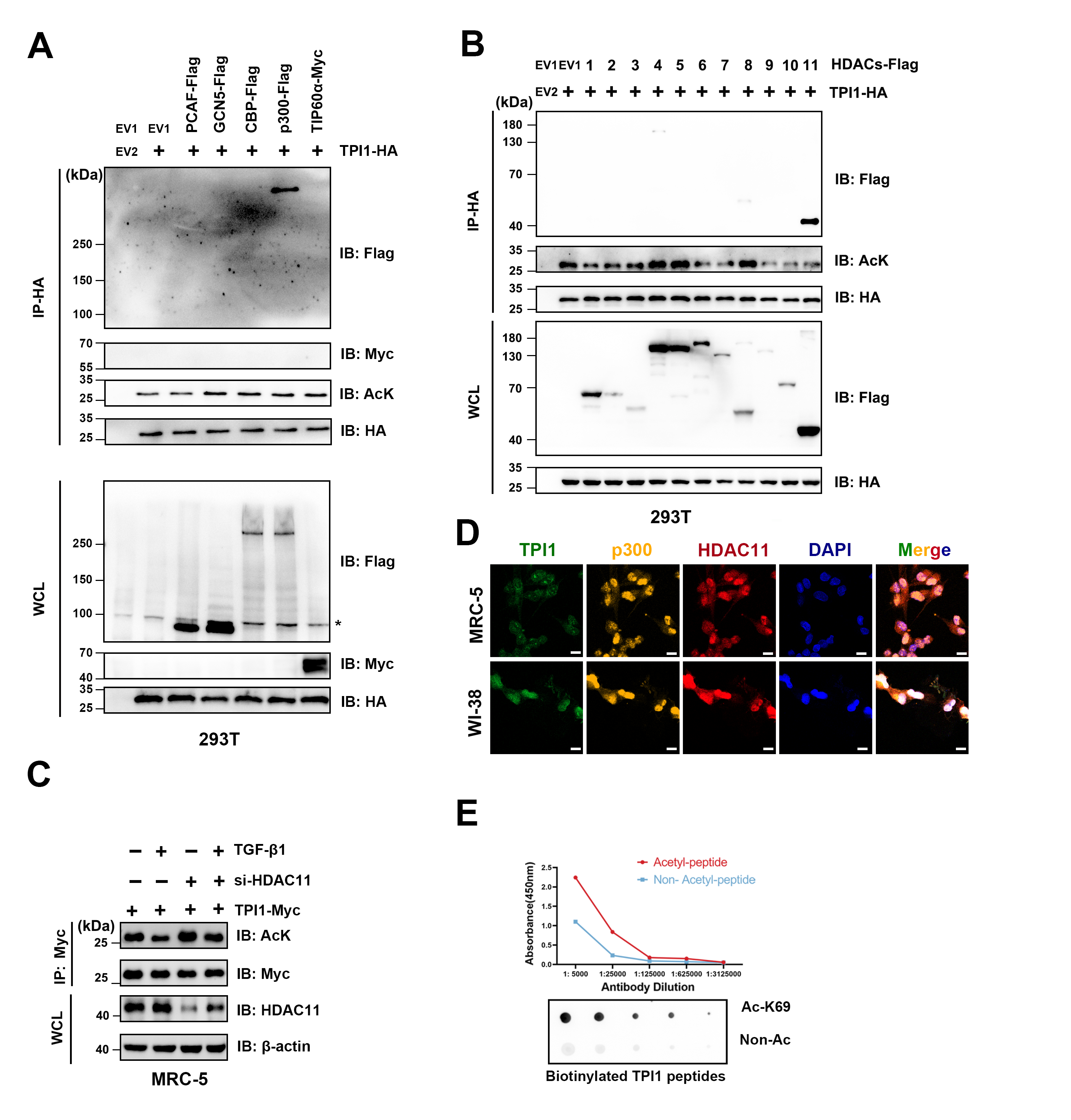

Supplement: Supplementary 1 — Graphical Abstract Figs. S1 to S4 Table S1 [file research.0953.f1.zip › FigureS3.tif]

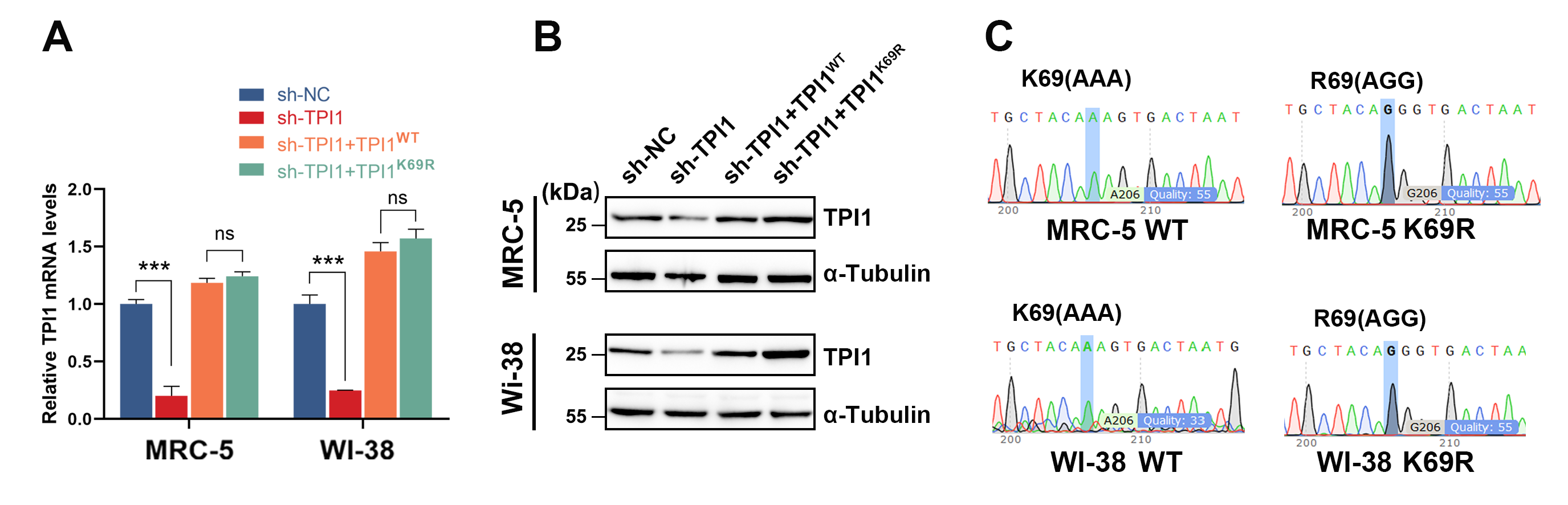

Supplement: Supplementary 1 — Graphical Abstract Figs. S1 to S4 Table S1 [file research.0953.f1.zip › FigureS4.tif]

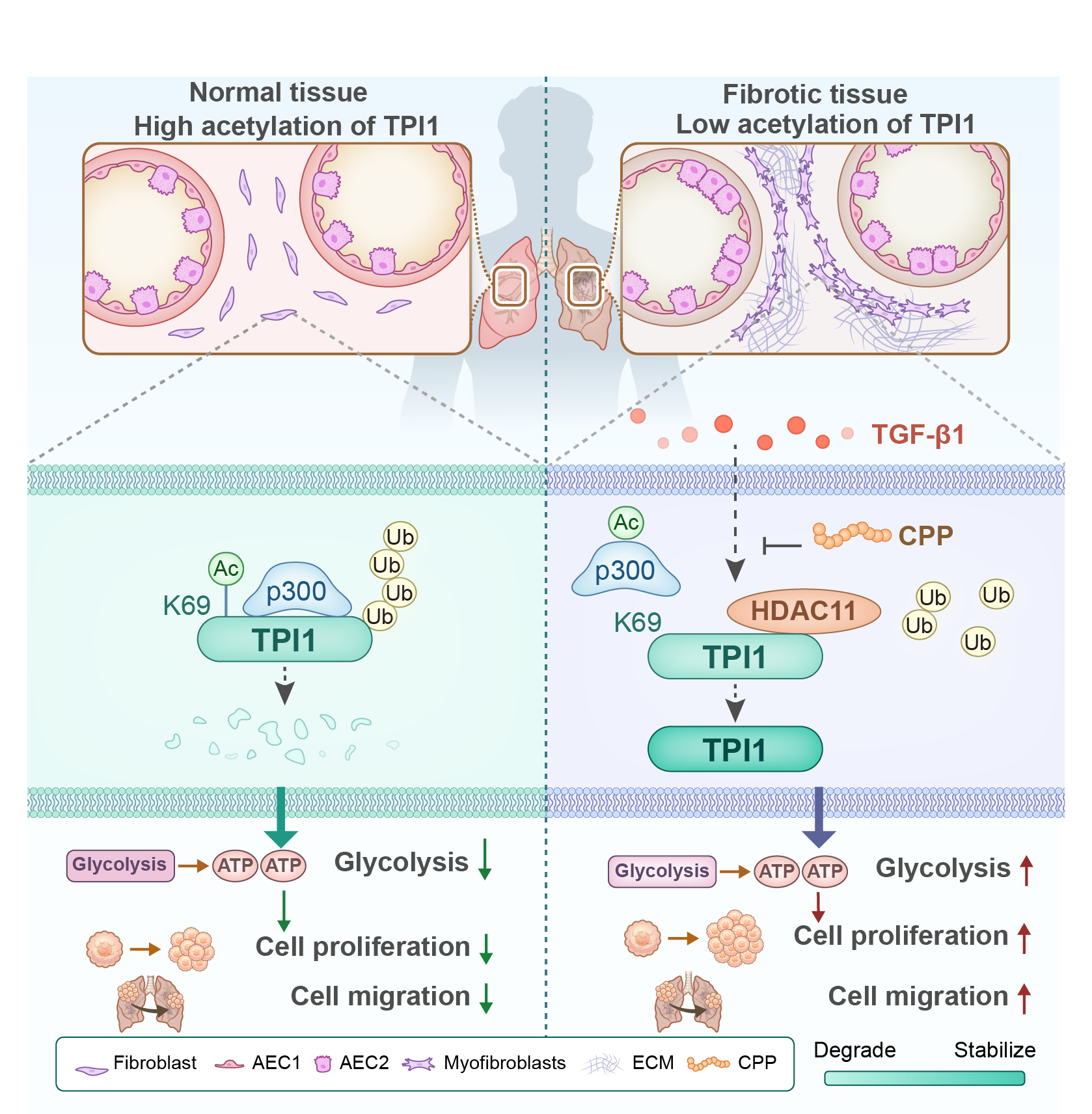

Supplement: Supplementary 1 — Graphical Abstract Figs. S1 to S4 Table S1 [file research.0953.f1.zip › Graphical abstract.png]
